# Predictive Modeling of Compression Strength of Waste PET/SCM Blended Cementitious Grout Using Gene Expression Programming

**DOI:** 10.3390/ma15093077

**Published:** 2022-04-23

**Authors:** Kaffayatullah Khan, Fazal E. Jalal, Mudassir Iqbal, Muhammad Imran Khan, Muhammad Nasir Amin, Majdi Adel Al-Faiad

**Affiliations:** 1 Department of Civil and Environmental Engineering, College of Engineering, King Faisal University (KFU), Al Ahsa 31982, Saudi Arabia; mgadir@kfu.edu.sa; 2 Shanghai Key Laboratory for Digital Maintenance of Buildings and Infrastructure, State Key Laboratory of Ocean Engineering, School of Naval Architecture, Ocean & Civil Engineering, Shanghai Jiao Tong University, Shanghai 200240, China; jalal2412@sjtu.edu.cn (F.E.J.); mudassiriqbal29@sjtu.edu.cn (M.I.); 3 Department of Civil Engineering, University of Engineering and Technology, Peshawar 25120, Pakistan; 4 Department of Civil & Environmental Engineering, Universiti Teknologi PETRONAS, Seri Iskandar 32610, Malaysia; muhammad_17007177@utp.edu.my; 5 Department of Chemical Engineering, College of Engineering, King Faisal University (KFU), Al Ahsa 31982, Saudi Arabia; malfaiad@kfu.edu.sa

**Keywords:** waste PET, supplementary cementitious materials, compressive strength, artificial intelligence, GEP

## Abstract

The central aim of this study is to evaluate the effect of polyethylene terephthalate (PET) alongside two supplementary cementitious materials (SCMs)—i.e., fly ash (FA) and silica fume (SF)—on the 28-day compressive strength (CS_28d_) of cementitious grouts by using. For the gene expression programming (GEP) approach, a total of 156 samples were prepared in the laboratory using variable percentages of PET and SCM (0–10%, each). To achieve the best hyper parameter setting of the optimized GEP model, 10 trials were undertaken by varying the genetic parameters while observing the models’ performance in terms of statistical indices, i.e., correlation coefficient (R), root mean squared error (RMSE), mean absolute error (MAE), comparison of regression slopes, and predicted to experimental ratios (ρ). Sensitivity analysis and parametric study were performed on the best GEP model (obtained at; chromosomes = 50, head size = 9, and genes = 3) to evaluate the effect of contributing input parameters. The sensitivity analysis showed that: CS_7d_ (30.47%) > CS_1d_ (28.89%) > SCM (18.88%) > Flow (18.53%) > PET (3.23%). The finally selected GEP model exhibited optimal statistical indices (R = 0.977 and 0.975, RMSE = 2.423 and 2.531, MAE = 1.918 and 2.055) for training and validation datasets, respectively. The role of PET/SCM has no negative influence on the CS_28d_ of cementitious grouts, which renders the PET a suitable alternative toward achieving sustainable and green concrete. Hence, the simple mathematical expression of GEP is efficacious, which leads to saving time and reducing labor costs of testing in civil engineering projects.

## 1. Introduction

Plastic production and increased usage have led to significant environmental damage. Plastic waste generation is increasing rapidly with increases in the global population, and it is predicted that this worldwide generation may be doubled by 2050 [1]. An exponential increase in the production of plastics has been reported such that, during 1950 and 2015, it increased from 2.3 million tons to 448 million tons [2]. Another study showed that about half of all plastic has been produced in the last 15 years. Of this plastic waste, approximately 8 million tons have been recorded as washing into the oceans every year [2]. It has been estimated that more than 8.3 billion tons of plastic has been manufactured since 1950s and around 60% of that plastic has ended up in landfills and the natural environment [3]. Therefore, to cope with this global environmental issue, it is immensely pertinent to explore the recycling of waste plastics. In this scenario, replacement of virgin materials used in construction industries with waste materials is desideratum [4]. One such alternative is recycling and reusing waste plastic in the construction of roads/highways. Waste plastic has widely been used as a substitute for bitumen or as an aggregate replacement in asphalt concrete pavements. Significant improvement in the performance properties of binder and asphalt mixtures has been reported previously [5,6]. A significant contribution to the economy and environment can be attained by recycling single-use waste plastic on a wide scale.

Recycling of waste polyethylene terephthalate (PET) in cementitious materials and its effect on mechanical properties have also been investigated in a few studies. In the concrete industry, waste plastics are used as a fiber or replacement of fine and coarse aggregates [7]. They have been used to replace aggregates in order to produce lightweight concrete elements [8]. Studies have shown an increase in the tensile strength and crack resistance of concrete by inclusion of recycled PET as a fiber in the concrete mixture [9,10]. However, the incorporation of PET as an aggregate or sand replacement with a volume of more than 10% causes a significant reduction in compressive strength compared with that of normal strength concrete [11,12]. Moreover, partial replacement of cement with plastic waste has also been studied, where significant reduction in the compressive strength was noticed. The reduction in compressive strength may reach up to 23% and 72% at 5% and 20% replacement of the cement, respectively [13]. Schaefer et al. [14] also used waste PET as a partial replacement of cement in mortars and observed reduction in compressive strength. However, using gamma irradiated PET instead of regular PET and additional replacement of FA or SF in cement mortar resulted in improved strength properties [14]. Some other studies also reported that the addition of gamma irradiated waste plastic ameliorated the strength properties of cementitious grouts [15,16,17,18]. 

The utilization of natural resources can be reduced while using waste plastic instead of aggregates or sand, which in return reduces environmental pollution and the cost of construction alongside contributing to better engineering properties of asphalt concrete [19]. Moreover, recycling waste plastic would reduce the burden of landfilling and save marine life [20]. Furthermore, industrial wastes and/or byproducts (such as FA and SF) are increasing due to increase in populations and industrialization all over the world. Due to shortage of land, many countries are facing problems of waste disposal which is a serious concern affecting the ecosystem. Therefore, reuse and recycling of these wastes and/or byproducts in the construction industries could be a viable alternative [21]. The perpetual and rapid utilization of natural resources is polluting the environment and deteriorating the surrounding environment [22]. FA and SF are categorized as supplementary cementing materials (SCM) owing to their pozzolanic properties, and therefore they contribute to the improvement in performance properties of cement concrete. The use of materials or mineral admixtures to replace cement in mortar and concrete is almost unavoidable in the effort to achieve sustainability, superior performance, and financial benefits [23,24]. Therefore, the recycling of municipal wastes (such as glass, plastic, rubber, wood, etc.) and industrial wastes/byproducts (such as FA, SF, ground granulated blast-furnace slag, etc.) as replacements for cement, sand, or aggregates could be beneficial to the environment in terms of reducing the usage of non-renewable natural resources and to the construction industry in terms of cost and enhancing the properties of concrete [21,25].

Artificial Intelligence (AI) approaches are becoming increasingly popular as a result of their improved predictive capability opposed to earlier techniques, and they are being utilized to simulate the complicated behavior of a range of structural engineering problems [26]. Data mining for processes in chemistry, materials, and especially civil engineering, has been frequently documented since 2000, which is largely attributed to the rapid growth of machine learning (ML) algorithms [27,28,29]. In the past few years, commonly deployed AI techniques employing conventional statistical methods in civil engineering include, artificial neural networks (ANNs) [30], genetic algorithm (GA) [31], genetic expression programming (GEP) [32], multi expression programming (MEP) [33], support vector machines (SVMs) [34], alternate decision trees (ADTs) [35], ensemble random forest (RF) regression [36], etc. Note that Giustolisi and Doglioni [37] advocated using white, black, and grey colors to categorize distinct mathematical models. The known variables and parameters of the first kind—the white-box model—are based on physical laws, which build correct physical connections and provide maximum transparency. Second, black-box models rely on regressive data-driven systems with unknown functional forms of connections among variables that must be approximated (e.g., ANN, ANFIS, etc.). Finally, grey-box models represent logical systems where a mathematical framework better assesses the behavior of the system (e.g., GEP, MEP, etc.). GEP can be classified as a ‘grey-box model’ because of its conceptualization of physical phenomena in a symbolic and easy way [38,39,40]. The GEP-based models are found to yield better results in contrast to other genetic-based and ANN-based approaches [29,41,42]. It is also associated with the fact that a typical GEP chromosome consists of head and tail that comprise particular symbols, thus providing a better way to encode syntactically correct computer programs as compared with the MEP approach [43]. In the past decade, GEP has been broadly and efficaciously used in addressing specific structural engineering issues, for instance, evaluating the compression strength of concrete incorporating (fly ash admixture, geopolymer, eco-efficient GGBS-based geopolymer, nanomaterials, etc.), the splitting tensile strength from the compressive strength [44], and the post-fire compressive strength of recycled PET aggregate concrete reinforced with steel fibers [45], among others. The GEP approach is widely deployed in solving regressions, modeling functions, predicting, detecting, and data mining [46,47]. In addition, the formulated genetic programming models are efficient due to absence of the assumption of fixed connection for developing a GEP model [48]. 

Furthermore, Ferreira and Jalali [49] predicted the early age compressive strength using a technique based on the nondestructive testing data. According to [50], models based on multi-layer feed-forward neural networks (MFNNs) may be built to forecast concrete’s 28-day compression strength on the basis of its selective influencing parameters. Rafi and Nasir [51] suggested an analytical approach for predicting concrete’s 28-day strength (CS_28d_) based on its 7-day strength (CS_7d_) by proposing a mathematical equation which was further expanded to forecast the strength of concrete constructed using Pakistani cements. To date, no attempts were made by previous researchers to formulate empirical models using the GEP approach for forecasting the 28-day compression strength (CS_28d_) of waste PET/SCM blended cementitious grout despite of their practical significance. With these uncertainties in consideration, the present research incorporates the GEP method to compute the compression strength of waste PET/SCM blended cementitious grout to compare the behavior of a variety of formulated GEP models.

## 2. Materials and Methods

### 2.1. Materials

Materials used in the experimental program were collected from local vendors, which include ordinary Portland cement, waste PET, SF, FA, and superplasticizer. Waste PET (particle size less than 150 µm) was used as a replacement of cement. The waste PET was obtained from a plastics factory, where PET flakes are recycled into plastic-based products. This process of recycling PET results in a sufficient amount of powder being discarded for disposal. For use in cementitious grout, the PET powder obtained was sieved so that the particle size was less than 150 microns. Table 1 shows the basic characteristics of waste PET.

### 2.2. Cement Grout Preparation and Testing Methods

The mixing of cementitious grouts were performed in accordance with ASTM C305 [52]. The required quantity of cement, waste PET, and SCMs (i.e., FA and SF) were initially dry mixed followed by adding two-thirds water and further mixing. The remaining water and superplasticizer were added and mixed to ensure the homogeneity of cement grouts. The mixed proportion of grouts containing PET and SCM are presented in Table 2.

The fresh cement grout was checked for the flow value using Malaysian flow cone apparatus [53]. According to specifications, 11–16 s are required for 1 L of fresh grout to flow-out of the cone [53]. The purpose of achieving such flow value (or flowability) is required to ensure proper filling of voids in an open-graded asphalt mixture. Moreover, mold (50 × 50 × 50 m^3^) were also prepared from all combination of grouts and tested for evaluating the compressive strength. After demolding, the specimens were kept in water bath for curing until test date. On completion of curing period (i.e., 1, 7, and 28 d), specimens were tested using a universal testing machine (UTM) with a capacity 3000 kN and at a rate of 0.90 kN/second for the determination of compressive strength (CS_1d_, CS_7d_, CS_28d_, respectively) of hardened cement grouts following ASTM C109 [54]. The values of the input and output parameters are given in Table S1 (Supplementary Materials).

### 2.3. Overview of GEP Method

**Figure 1:**
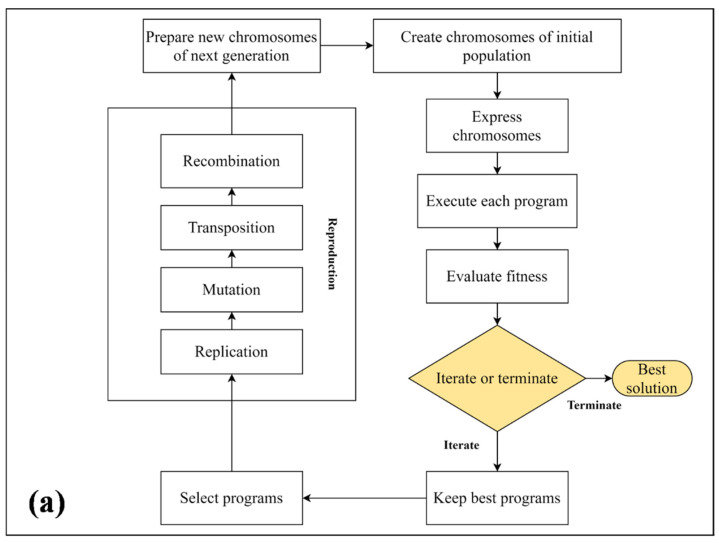

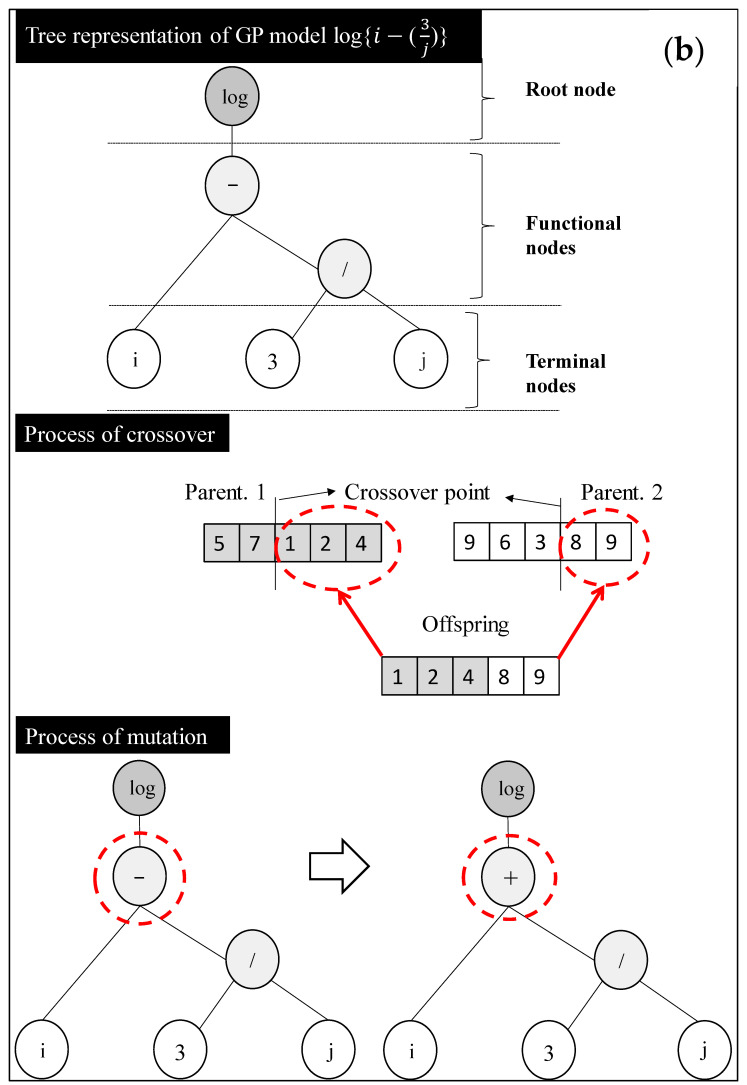
(**a**) Basic working process of gene expression programming (GEP) [64]. (**b**) Expression tree, crossover, and mutation processes in a representative GEP model [42].

A GEP approach based on Darwin’s evolution theory and Mendel’s genetic theory is one of the most intellectually compelling computational intelligence approaches [55,56]. With GEP, there are two languages: the language of genes and the language of expression trees (ETs), and understanding the sequence or structure of one is equivalent to understanding the other [57]. A typical GEP model involves the following steps, as shown in Figure 1a.

- In GEP modeling, chromosome numbers are randomly generated for designated numbers, and the Karva language (which represents symbols) is used for introducing the chromosomes. The chromosomes and genes typically consist of a head and a tail. A head consists of either a function or a set of terminal symbols, while a tail consists of only terminal symbols. A model’s head size depends on the complexity of each parameter, whereas the number of genes determines the number of sub-ETs.
- The length of chromosomes is fixed, so the chromosomes can be transformed into an algebraic expression [58] as illustrated in Figure 1b. GEP genes contain a fixed list of terms, so each term represents a function; for example, arithmetic operations (+, −, ×, /), Boolean logic functions (AND, OR, NOT, etc.), mathematical functions (cos, sin, ln), conditional functions (IF, THEN, ELSE), or other function categories [59].
- The chromosomes are then represented by ETs, which come in diverse shapes and sizes. After that, primary genetic operators—such as crossover, mutation, transposition, and gene recombination (1-point, 2-point, and gene recombination)—are applied to each chromosome according to their ratios [60]. Figure 1b depicts a typical expression tree (ET), illustrating how crossovers and mutations work. In Equation (1), we can also see that the ET is expressed by means of a Karva notation or K-expression (Figure 1b) [61].
  (1) $${\mathrm{ET}}_{\mathrm{GEP}}=log\left(i-\frac{3}{j}\right)$$

Once the stopping condition (highest number of generations or best solution) is reached, the process is complete [62].

d. When the maximum number of iterations or favorite fitness value is not achieved, the roulette wheel method (as well as greedy over the selection, ranking selection, tournament selection, and elite selection [27]) is adopted, which selects the viable chromosomes of first-generation for continuation to the next. Herein, the process is rewound for a specified number of generations or until the selection of the optimal solution is made [63].

## 3. Results and Discussion

### 3.1. Effect of Genetic Parameters on Performance of Models

**Figure 2:**
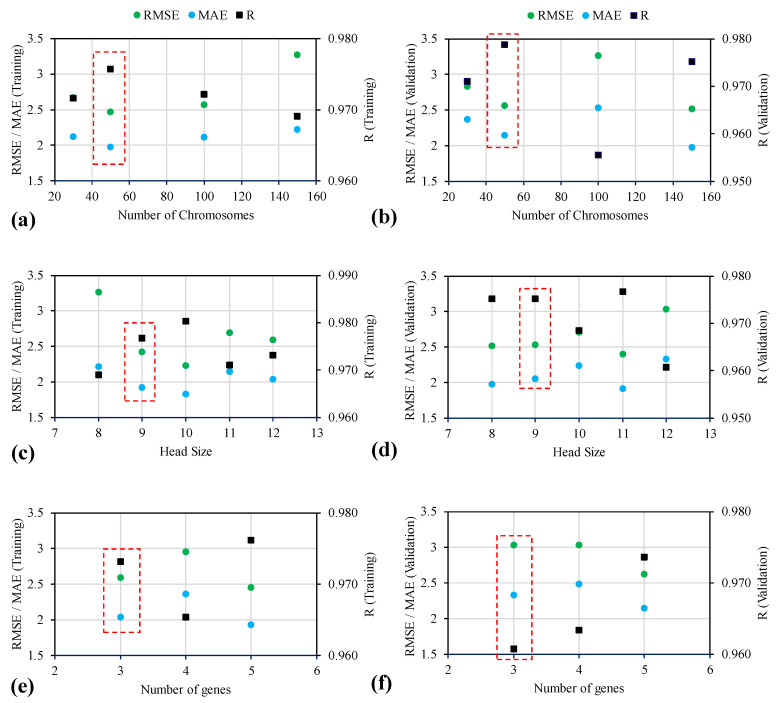
Evaluating the effect of genetic parameters on the performance of models (MAE, R, RMSE) for the case of (**a,b**) number of chromosomes, (**c,d**) head size, and (**e,f**) number of genes.

While exercising modeling using the GEP model, genetic parameters—namely, number of chromosomes, head size, and number of genes—were varied alongside the evaluation of models in the form of statistical equations for the correlation coefficient (R), mean absolute error (MAE), and root mean square error (RMSE) given as Equation (2) to Equation (4) in accordance with the previous research [65,66,67,68,69,70]. Initially, number of chromosomes were changed from 30 to 150 to select the best number of chromosomes for the next model generation. The best model for variable setting chromosomes was based on the overall MAE and R^2^ for the developed models. Subsequently, head size was changed between 8 and 10. Finally, number of genes was varied from 3 to 5. Further increase in the number of genes may affect the performance of the model; however, it also complexifies the mathematical equation. Figure 2 displays the effect of setting parameters on the performance of the models. Figure 2a,c,e and Figure 2b,d,f depict the performance of the models for the training data and validation data with respect to number of chromosomes, head size, and number of genes, respectively. It can be observed that increasing the number of chromosomes from 30 to 50 increased the performance in the sense of increasing R and decreasing MAE and RMSE for both the training and validation data. In contrast, an additional increase in the number of chromosomes from 50 to 100 decreased the performance of the models. The best number of chromosomes was achieved at 50; therefore, more trials on developing the best models by changing head size were exercised with 50 chromosomes. 

To see the impact of changing head size on the performance of the developed models, it can be seen that increasing the head size from 8 to 9 increased the values of R from 0.969 to 0.977 for the training data; whereas, for the validation data, the value of R remained unchanged for the 8 and 9 head size. Similarly, the values of RMSE and MAE decreased from 3.267 and 2.218 MPa to 2.432 and 1.918 MPa for the training data. The counterpart values of the validation data somehow increased; however, based on overall value of R, the trials carried out with the 9 head size performed better. Trails 7, 8, and 9 were interpreted with comparatively lower performance. The variation in number of genes from 3 to 5 did not yield the fruitful performance of the model. In summary, the best setting parameters in this comprehensive exercise were obtained as 50, 9, and 3 number of chromosomes, head size, and number of genes, respectively.

(2) $$\mathrm{MAE}=\frac{\sum_{k=1}^{m}\left|E_{k}-P_{k}\right|}{m}$$

(3) $$\mathrm{RMSE}=\sqrt{\frac{\sum_{k=1}^{m}{\left(E_{k}-P_{k}\right)}^{2}}{m}}$$

(4) $$R=\frac{\sum_{k=1}^{m}\left(E_{k}-{\bar{E}}_{k}\right)\left(P_{k}-{\bar{P}}_{k}\right)}{\sqrt{\sum_{k=1}^{m}{\left(E_{k}-{\bar{E}}_{k}\right)}^{2}\sum_{k=1}^{m}\left(P_{k}-{\bar{P}}_{k}\right)}}$$

In the above equations, $E_{k}$ and $P_{k}$ represent the actual observations and estimated outcomes, respectively; ${\bar{E}}_{k}$ and ${\bar{P}}_{k}$ denote the average of the actual observations and estimated outcomes, respectively; and m is the total number of records.

### 3.2. Prediction Performance of Models

#### 3.2.1. Statistical Evaluation 

Five input parameters (PET, SCM, Flow, CS_1d_, and CS_7d_) were selected in the current study for determination of the output parameter (i.e., CS_28d_). Although the parameters affecting the strength of cement-based materials are considered to be material mixing ratios—such as the water/binder ratio and the ratio of fine aggregates used—are also desideratum; however, these parameters were kept constant in this study, whereas the aforementioned parameters were varied. The prediction was made based on the varying parameters and this study can be extrapolated in future regarding CS prediction of PET/SCM blended cementitious grout for the design mix used in the study as presented in Section 2.2. The formulation of CS_28d_ of waste PET/SCM blended cementitious grout is given by Equation (5) below.

(5) $${\mathrm{CS}}_{28d}=f\left(\mathrm{PET},\mathrm{SCM},\mathrm{Flow},{\mathrm{CS}}_{1d},{\mathrm{CS}}_{7d}\right)$$

Pearson’s correlation coefficient, abbreviated as r, is amongst the most widely employed metrics of depicting relationship [71]. Interdependency as well as multicollinearity are better recognized to be examined since they present issues with the understanding of a given AI model [72]. Therefore, the Pearson’s correlation matrix was computed for the experimental database of waste PET/SCM blended cementitious grout considered here which comprise five input parameters (PET, SCM, Flow, CS_1d_, and CS_7d_) for determination of the output parameter (i.e., CS_28d_), as shown in Table 3. The correlation matrix is defined with the help of a square symmetrical H × H matrix such that the (uv)th element equals the correlation coefficient Ruv among the (u)th and the (v)th variable. Note that the diagonal members (correlations of the considered input parameters with each other) are always 1 [42,73]. The r varies from −1 and 1 (0 means zero correlation, while, ±1 depicts strong positive and negative correlation, respectively). The correlation degree follows the order: CS_7d_ > CS_1d_ > PET > SCM > Flow (r = 0.93, 0.85, −0.67, 0.64, and −0.52, respectively) which indicates a major contribution of CS_7d_, CS_1d_, and PET on the CS_28d_ of waste PET/SCM blended cementitious grout. Since the r values of all parameters are exceedingly higher, they were therefore considered while formulating the GEP models.

The summary of descriptive statistics of the input (PET, SCM, Flow, CS_1d_, and CS_7d_) and output parameter (CS_28d_) are given in Table 4. The minimum and maximum limits, standard deviation (SD), kurtosis, as well as skewness values for all these parameters in the considered database have been tabulated. A smaller value of SD suggests that the values are mostly nearing the average (PET, SF, and Flow), while a larger SD represents comparatively higher spread out (CS_1d_, CS_7d_) [74]. Skewness (value could be positive, zero, negative, or undefined) helps in determining the magnitude of asymmetry of the probability distribution in case of a real-valued arbitrary parameter from the standpoint of its average value [75]. Furthermore, kurtosis generally ranges between −10 (heavy-tailed) and + 10 (light-tailed), which helps in the elucidation of the shape of a probability distribution, as described by Brown and Greene [76]. The kurtosis values for PET, SF, CS_1d_, and CS_7d_ are negative and range between −0.3 and −1.6 (follow mesokurtic distribution), whereas the only positive value is obtained in case of Flow, i.e., 0.66 (follow leptokurtic distribution) [77,78].

Ten trials were run in order to determine the parametric combination with the overall R (average of training and validation values) and MAE value as well as for CS_28d_ of waste PET/SCM blended cementitious grout (Table 5). The details of these undertaken trials for identification of optimal combination of the GEP parameters yielding better performance are presented (Table 4). The GEP algorithm is kept running indefinitely so that the correlation, as well as fitness functions (RMSE in this case), do not alter their respective values. Furthermore, while an arithmetic function is selected to connect them, the genes alongside their corresponding head sizes are continuously increased (addition function is taken into consideration here). The process is performed numerous times until the best model is established. Table 6 shows the best settings for the GEP model utilized in this study. The drop recorded in model error is determined by the R value, that is connected to the inclusion of predictor parameters. Note that, the overall R was highest—i.e., 0.995— while overall MAE was 2.059 for Trial 2 at default settings (chromosomes = 30, head size = 8, genes number = 3). However, in case of Trial 5, the R value was 0.953 while its MAE was 1.9865, because of which the latter trial was considered to be the final optimized model. Further changes in the GEP settings resulted in lower R values, higher R^2^, and lower MAE.

#### 3.2.2. Comparison of the Regression Slopes between Experimental and Predicted Results

**Figure 3:**
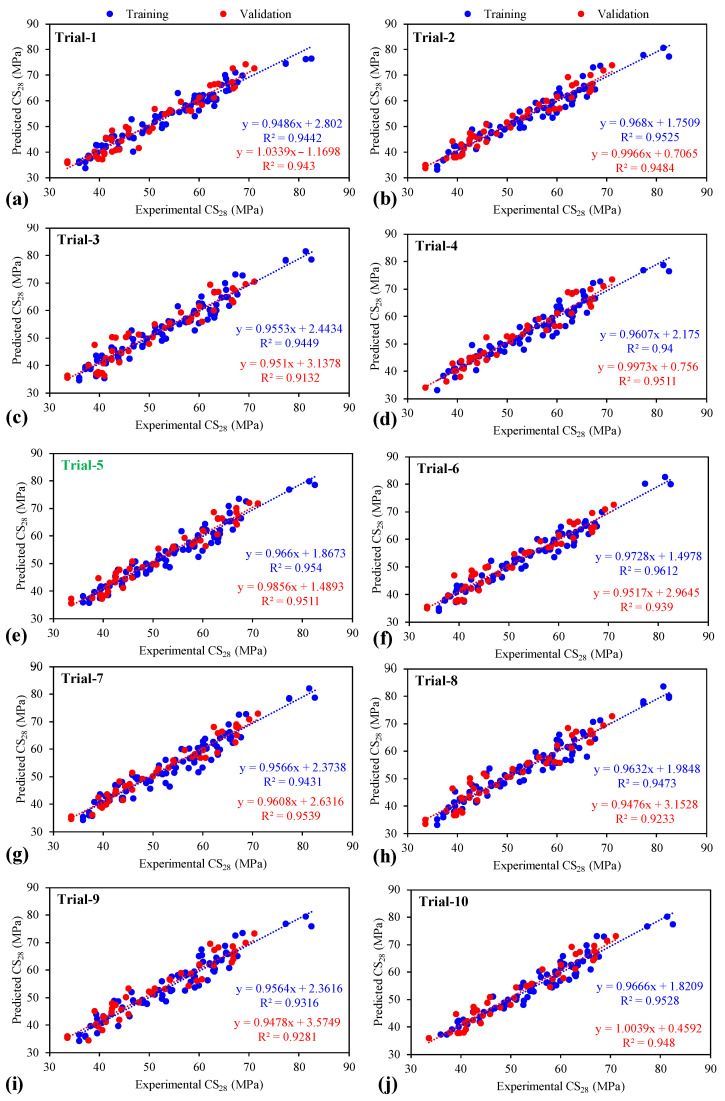
Comparison of the regression slopes between experimental and predicted results for all the (**a**–**j**) 10 GEP trials using different combinations of hyper parameters.

Figure 3a–j presents the comparison of the regression slopes between modeled and experimental results for all the 10 GEP trials (training and validation datasets are represented by blue and red, respectively). It can be seen in Figure 3 that the CS28 is accurately predicted for almost all the proposed models wherein the lowest–maximum regression slopes for training and testing datasets are 0.9468 (Trial 1)–0.9728 (Trial 6) and 0.947 (Trial 8)–0.9973 (Trial 4), respectively. In the previous studies, slopes ranging between 0.80 and 0.90 were considered indicators of the achievement of higher performance, when modeled with GEP approach [29,79,80]. Based on their findings, it can be stated that all of the formulated GEP trials exhibited comparatively better performance. Trial 5 was selected as the optimal trial on the basis of overall R and MAE values (in the previous section). In addition, the nearness of the points to the ideal fit (1:1 line) and the presence of the majority of the points inside the allowable confidence interval elucidates the validity of GEP models [42]. The points which are closer to the regression line depict better prediction performance of a formulated GEP model [81]. While considering the plots of regression slopes (in Figure 3), it can be observed that Trial 6 and Trial 4 possessed the maximum slopes for training and validation datasets, respectively. On the contrary, the values of slope in the case of Trial 5 were recorded as 0.957 (training) and 0.961 (validation). Upon comparing the overall MAE values of the three trials, it is found that Trial 5 has the lowest value (1.9865) in contrast to Trial 4 (2.096) and Trial 6 (2.0325). Moreover, the comparatively higher R value (0.953) in case of Trial 5 further makes it a final choice to be considered as the most optimal model. Note that, an R value exceeding 0.8 illustrates better performance of the GEP model [79,82]. Therefore, the prediction accuracy of Trial 5 is higher.

#### 3.2.3. Predicted to Experimental Ratio (ρ)

**Figure 4:**
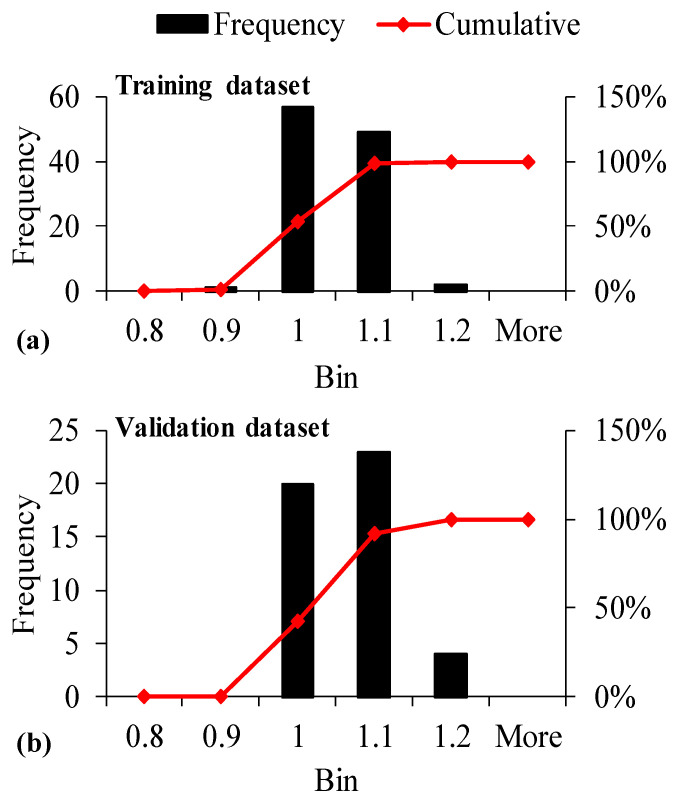
Histogram analysis of predicted to experimental ratios (ρ) to determine CS_28_ for GEP Trial 5 in case of (**a**) Training dataset, and (**b**) Validation dataset.

A variety of statistical methods are used to investigate the performance of developed models. The values obtained from the predicted models divided by the experimental results—expressed as predicted-to-experimental ratio (ρ)—is also employed to evaluate the performance of the developed model [83]. The ratios obtained from dividing predicted results by experimental values are plotted in the form of Table 7, taking the bin range as 0.2. It can be observed that all models interpreted 100% ρ values in between 0.8 and 1.2. This suggests that the accuracy of all the developed models is within ±20% error. The results of ρ for Trial 5—which is concluded as the best model achieved from evaluation in terms of statistical indices and comparison of regression slopes—was plotted in the form of a histogram, decreasing the bin range from 0.2 to 0.1 to see the accuracy of the respective model in a closer view shown in Figure 4. ρ values of 98.17% and 91.49% o were obtained within 0.9 to 1.1 for the training and validation datasets, respectively. This manifests the model error within ±10%, reflecting a more robust performance for the selected optimum model.

#### 3.2.4. GEP Formulation

**Figure 5:**
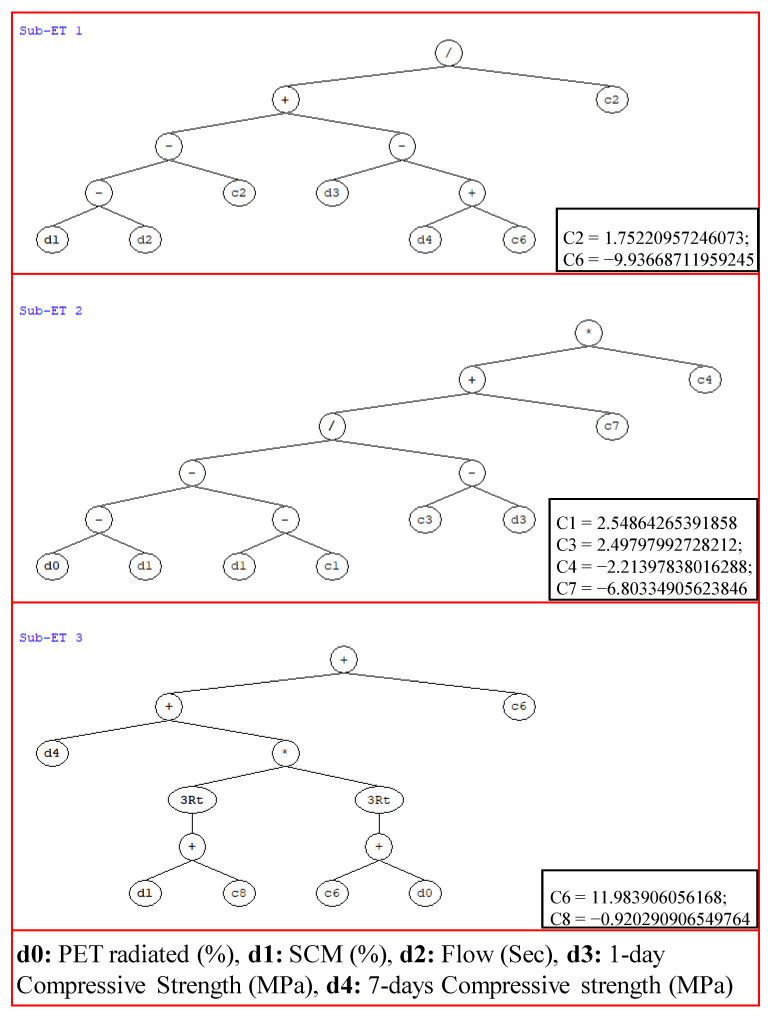
GEP tree obtained from GeneX pro tool for the most optimal case (Trial 5) for determining simple mathematical expression of compression strength of waste PET/SCM blended cementitious grout.

The expression trees shown in Figure 5 and the MATLAB code obtained from the GEP model were used to extract mathematical equation (Equation (6)) to predict the compressive strength of PET containing SCM based grout in accordance with the previous literature [84]. The prediction equations were obtained for the best model, Trial 5, in this study. The complexity of the mathematical equation depends upon the number of sub-ETs which depends on the number of genes. It is evident that Trial 5 contains three genes in its setting parameters; hence, four sub-ETs can be observed in Figure 5. Furthermore, Figure 5 shows linking functions (+, −, /, ×, cubic root), constants (C1 to C8), and variables in the form of d0 to d4 denoting PET percentage, percentage of SCM, flow, 1-day compressive strength, and 7-day compressive strength of cementitious grout, respectively, used in the formulation of GEP mathematical model.

(6) $$CS_{28d}=\left(\left(\left(\left(X_{1}-X_{2}\right)-1.75\right)+\left(X_{3}-\left(X_{4}-9.94\right)\right)\right)/1.75\right)+\left(\left(\left(\left(\left(X_{0}-X_{1}\right)-\left(X_{1}-2.55\right)\right)/\left(2.55-X_{4}\right)\right)-6.8\right)*\left(-2.2\right)\right)+\left(X_{4}+\left(\sqrt[{3}]{X_{1}-0.92}\right)*\sqrt[{3}]{\left(11.98+X_{0}\right)}\right))+11.98)$$

where, *X*_0_ = PET, *X*_1_ = SCM, *X*_2_ = Flow, *X*_3_ = CS_1d_, and *X*_4_ = CS_7d_.

### 3.3. Sensitivity and Parametric Analysis

It is critical to conduct a number of assessments over AI formulated models to guarantee that the suggested models possess robustness and that they perform well for different unseen data. Sensitivity and parametric tests demonstrate their robustness [72,85]. The sensitivity analysis (SA) on the entire database determines how susceptible a constructed model is to changes in the variables under consideration [79,86]. The relative contributions of the input factors (PET, SCM, Flow, CS_1d_, and CS_7d_) are taken into consideration here to forecast the CS_28d_ of waste PET/SCM blended cementitious grout by performing the SA. 

For a particular input variable, Si, the SA can be computed using the Equations (7) and (8), respectively (such that *t* = 156, in the present study).

(7) $$t_{i}=f_{max}\left(S_{i}\right)-f_{min}\left(S_{i}\right)$$

(8) $$SA\;\left(\%\right)=\frac{T_{i}}{\sum_{n}^{j=1}T_{j}}*100$$

where fmax (*S_i_*) and fmin (*S_i_*) denotes, respectively, the maximum and minimum of forecasted CS_28d_ on the basis of the *i*th input domain, whereas the rest of the input variables remain constant at their mean.

The SA value ranges from 0 to 1, indicating the relative contribution of each input variable to the predicted output compression strength (SA = 1 indicates higher impact, SA = 0 indicates least effect) [87,88]. 

**Figure 6:**
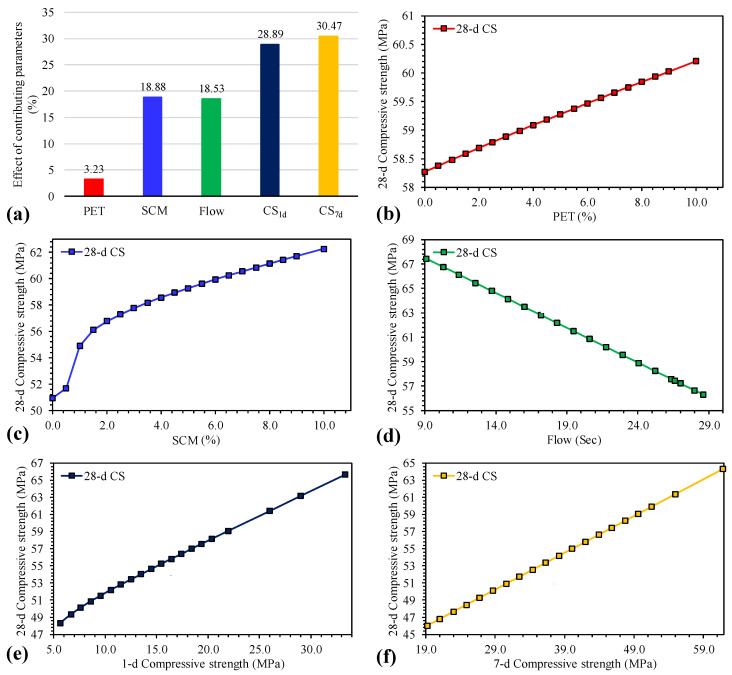
(**a**) Sensitivity analysis and (**b**–**f**) parametric study of all the considered input parameters (i.e., PET, SCM, Flow, CS_1d_, and CS_7d_, respectively).

Firstly, the results of sensitivity analysis in Figure 6a shows a rising trend of input parameter contributions to determine the CS_28d_ of waste PET/SCM blended cementitious as CS_7d_ (30.47%) > CS_1d_ (28.89%) > SCM (18.88%) > Flow (18.53%) > PET (3.23%). Therefore, CS_7d_ is most substantial factor that influences the long term CS_28d_ of cementitious grout mixes, followed by CS_1d_, flow, and proportion of the SCMs. 

Secondly, the parametric study in Figure 6b–f shows that with an increase in the dosage levels of the PET (0–10%), proportioning of SCM (0–10%), CS_1d_, and CS_7d_ led to a positive growth in the CS_28d_ of cementitious grout mixes. Generally, the increase in PET leads to a decrease in the compression strength; however, in the current study, it was found that with increasing PET the CS_28d_ increased very slightly (up to 3.61%). This can be attributed to the inclusion of the SCMs (i.e., fly ash, silica fume, and metakaolin). It is pertinent to mention that when the percentage of recycled PET fiber is higher, the slump test and compressive strength values decreased [89]. Generally, the inclusion of plastic fragments to concrete reduces the fresh and dry density, thus decreasing the weight of the generated concrete [90]; however, since SCMs were used in conjunction, the CS_28d_ of cementitious grout mixes increased with higher dosages of PET. In addition, the cementitious materials (fly ash as well as silica fume) compensate the loss of CS_28d_ due to addition of the PET content [91]. Inclusion of PET fibers alongside silica fume-cemented sand increases the unconfined compression strength, as well as the energy absorption capacity [92] because silica fume tends to decrease the detrimental consequences on strength characteristics while increasing the compression strength [93,94]. On the other hand, the increase in flow values negatively impacted the CS_28d_ of cementitious grout mixes, which agrees with the findings of the past researchers—i.e., when the slump flow of concrete decreases, the CS increases [95,96,97,98].

## 4. Conclusions

This study aims to determine the impact of PET/SCM (polyethylene terephthalate (PET) and two supplementary cementitious materials (SCMs)—i.e., fly ash (FA) and silica fume (SF)—on the 28-day compressive strength (CS_28d_) of cementitious grouts using GEP. A new predictive model in the form of a simple mathematical expression has been formulated to compute the CS_28d_ of cementitious grout mixes using easily determinable input parameters, such as, PET, SCM, flow, CS_1d_, and CS_7d_. Based on this investigation, the following conclusions can be drawn:

1. Ten GEP trials were conducted, and several performance indices were recorded for each trial. While investigating the effect of varying genetic setting parameters (chromosomes, head size, genes) to evaluate the CS_28d_ of cementitious grout mixes, it was noticed that the best hyper parameter for a particular GEP model strongly depended on trial and access method. The optimal genetic parameter for one GEP model for computation of CS_28d_ may be completely different from the optimum parameter for another model. Depending on variety of indices, the best GEP model was obtained for number of chromosomes = 50, head size = 9, and number of genes = 3.

2. The optimum statistical indices obtained in the case of the finally selected optimal model (Trial 5) were obtained as RMSE (2.423 and 2.531), MAE (1.918 and 2.055), and R (0.977 and 0.975) for training and validation datasets, respectively. In addition, the MAE values depict 3.6% (training) and 3.8% (validation) averaged error in the developed model. These values are significantly lower, which indicates accuracy and robust performance in terms of experimental and modeled values of the CS_28d_ of cementitious grout mixes in the formulated GEP model. 

3. The GEP model performance was further supplemented with the help of other statistical evaluating indices, such as: (i) slope of regression line between experimental and predicted results; and (ii) predicted to experimental ratios (ρ) for all the models. It it worth noting that the best model yielded regression slopes of 0.966 (training) and 0.985 (validation), which are more proximal to unity (i.e., ideal slope). The ρ values for all the 10 trials interpreted 100% of the values lying within ±20%. Furthermore, the optimal model resulted in 98.17% and 91.49% of the ρ values within ±10% error, which further confirms the final selection of the optimum model.

4. The MATLAB code extracted from the final GEP model was used to form a mathematical equation comprising easily determinable input parameters to evaluate the CS_28d_ of cementitious grout mixes, thus avoiding the laborious, time-consuming, and costly testing of the samples and thereby improving the cost-effectiveness of civil engineering projects.

5. The sensitivity analysis revealed that CS_7d_ is the most significant parameter which impacts the long term CS_28d_ of cementitious grout mixes followed by CS_1d_, flow, and proportion of the SCM. Alongside incorporating the different SCMs, it is imperative to mention that the negative influence of the PET dosage on CS_28d_ was substantially neutralized (depicting only a slight increase). Moreover, the parametric study revealed that increasing the PET and proportioning of SCM (0 to 10% each), CS_1d_, and CS_7d_ resulted in a positive increase in the CS_28d_ of cementitious grout mixes. Conversely, the increase in flow values negatively impacted the CS_28d_. The current GEP model may be effectively deployed for future purposes to evaluate the 28-day compression strength of cementitious grout mixes.

## Figures and Tables

**Table 1:** Properties of waste PET (obtained from supplier).

Description	Values
Density (g/cm^3^)	1.35
Tensile strength (MPa)	187
Glass transition temperature (°C)	75
Melting point (°C)	250

**Table 2:** Mixed proportion of grouts containing PET and SCM.

Grout Type	Quantity (g)			
	Cement	PET	SCM (SF/FA)	Water
Control	3800	0	0	1330
5PET	3610	190	0	1330
10PET	3420	380	0	1330
5PET-5SCM	3420	190	190	1330
5PET-10SCM	3230	190	380	1330
10PET-5SCM	3230	380	190	1330
10PET-10SCM	3040	380	380	1330

**Table 3:** Pearson correlation matrix for the inputs and output parameters CS_28d_ of waste PET/SCM blended cementitious grout.

	PET	SCM	Flow	CS_1d_	CS_7d_	CS_28d_
PET	1					
SF	−4.495 × 10^−18^	1				
Flow	0.7411116	0.072472	1			
CS_1d_	−0.8411645	0.3140399	−0.528282	1		
CS_7d_	−0.7553707	0.5103415	−0.5603769	0.86217187	1	
**CS_28d_**	**−0.6680288**	**0.6363484**	**−0.5204947**	**0.84706071**	**0.9325534**	**1**

**Table 4:** Statistical evaluation of input and output parameters.

	PET (%)	SCM (%)	Flow (Sec)	CS_1d_ (MPa)	CS_7d_ (MPa)	CS_28d_ (MPa)
Min	0.00	0.00	9.10	5.64	19.19	33.64
Max	10.00	10.00	28.60	33.32	61.81	82.54
Mean	5.00	4.62	16.30	18.66	37.20	53.74
Median	5.00	5.00	15.40	17.91	36.54	54.18
SD	3.67	4.14	4.18	7.64	9.44	11.23
Skewness	3.3 × 10^−18^	1.5 × 10^−1^	9.5 × 10^−1^	2.3 × 10^−1^	4.6 × 10^−1^	3.0 × 10^−1^
Kurtosis	−1.3464	−1.5395	0.6675	−1.0730	−0.3549	−0.5814

**Table 5:** Summary of undertaken trials using GEP for evaluating the CS_28d_ of waste PET/SCM blended cementitious grout.

Trial No.	No. of Chromosomes	Head Size	Number of Genes	Constants Per Gene	Literals	Program Size	Training Dataset					Validation Dataset					Overall R
							Best Fitness	RMSE	MAE	R^2^	R	Best Fitness	RMSE	MAE	R^2^	R	
1	30	8	3	10	10	39	272.9	2.664	2.122	0.944	0.972	261.1	2.829	2.368	0.943	0.971	0.944
2	50	8	3	10	8	35	288.6	2.465	1.969	0.952	0.976	280.4	2.566	2.149	0.958	0.979	0.955
3	100	8	3	10	12	41	274.0	2.565	2.107	0.945	0.972	234.3	3.267	2.535	0.913	0.956	0.929
4	150	8	3	10	9	36	191.0	3.267	2.218	0.939	0.969	201.8	2.519	1.974	0.951	0.975	0.945
* 5	**50**	**9**	**3**	**10**	**11**	**41**	**292.1**	**2.423**	**1.918**	**0.954**	**0.977**	**283.2**	**2.531**	**2.055**	**0.951**	**0.975**	**0.953**
6	50	10	3	10	17	52	310.0	2.230	1.825	0.961	0.980	269.4	2.712	2.240	0.938	0.969	0.950
7	50	11	3	10	16	53	270.7	2.693	2.142	0.943	0.971	294.2	2.398	1.911	0.954	0.977	0.949
8	50	12	3	10	21	59	278.3	2.594	2.037	0.947	0.973	247.8	3.035	2.334	0.923	0.961	0.935
9	50	9	4	10	16	46	252.4	2.960	2.359	0.932	0.965	247.8	3.035	2.484	0.928	0.963	0.930
10	50	9	5	10	21	74	289.5	2.454	1.929	0.953	0.976	276.0	2.623	2.149	0.948	0.974	0.951

Note: The number of used variables in all the trials was 5. * Trial 5 is the most optimal trial.

**Table 6:** Parameters setting for GEP algorithm (Trial 5) to determine CS_28d_ of waste PET/SCM blended cementitious grout.

Parameters	GEP
Number of chromosomes	50
Number of genes	3
Head size	9
Linking function	Addition
Function set	+, −, ×, ÷, x^2^, x^1/3^
Maximum arity	2
Training records	109
Testing/validation records	47
Numerical constants	
Constants per gene	10
Type of data	Floating number
Maximum complexity	10
Ephemeral random constant	[–10, 10]
Genetic operators	
Strategy	Optimal evolution
Rate of mutation	0.00138
Function insertion	0.00206
Permutation	0.00546
IS and RIS transposition	0.00546
Inversion rate	0.00546
Uniform recombination	0.00755
Random and best cloning	0.0026
Constant fine tuning	0.00206

**Table 7:** Histogram analysis of predicted to experimental ratios (ρ) to determine CS_28_ for all the 10 GEP trials undertaken in the current study.

Dataset	Bin Ranges	Trial 1		Trial 2		Trial 3		Trial 4		Trial 5	
		Frequency	Cumulative (%)	Frequency	Cumulative (%)	Frequency	Cumulative (%)	Frequency	Cumulative (%)	Frequency	Cumulative (%)
Training	0.8	0	0.00	0	0.00	0	0.00	0	0.00	0	0.00
	1	54	49.54	56	51.38	54	49.54	52	47.71	58	53.21
	1.2	55	100.00	53	100.00	55	100.00	57	100.00	51	100.00
		Trial 6		Trial 7		Trial 8		Trial 9		Trial 10	
	0.5	0	0.00	0	0.00	0	0.00	0	0.00	0	0.00
	0.8	0	0.00	0	0.00	0	0.00	0	0.00	0	0.00
	1	55	50.46	53	48.62	50	45.87	51	46.79	57	52.29
	1.2	54	100.00	56	100.00	59	100.00	58	100.00	52	100.00
Validation		Trial 1		Trial 2		Trial 3		Trial 4		Trial 5	
	0.5	0	0.00	0	0.00	0	0.00	0	0.00	0	0.00
	0.8	0	0.00	0	0.00	0	0.00	0	0.00	0	0.00
	1	19	40.43	21	44.68	21	44.68	17	36.17	20	42.55
	1.2	28	100.00	26	100.00	25	97.87	30	100.00	27	100.00
		Trial 6		Trial 7		Trial 8		Trial 9		Trial 10	
	0.5	0	0.00	0	0.00	0	0.00	0	0.00	0	0.00
	0.8	0	0.00	0	0.00	0	0.00	0	0.00	0	0.00
	1	24	51.06	18	38.30	22	46.81	18	38.30	21	44.68
	1.2	22	97.87	29	100.00	25	100.00	29	100.00	26	100.00

## Data Availability

All the data utilized in this research are available on a reasonable request from the corresponding author.

## Supplementary Materials

The following supporting information can be downloaded at: https://www.mdpi.com/article/10.3390/ma15093077/s1, Table S1: Experimental database of five input parameters (PET, SCMs, Flow, CS_1d_ and CS_7d_) to evaluate the 28-d compression strength (CS_28d_) of waste PET/SCM stabilized cementitious grout.
