# Supplementary material for: Predictive Modeling of Compression Strength of Waste PET/SCM Blended Cementitious Grout Using Gene Expression Programming
Source: Materials (Basel). 2022 Apr 23;15(9):3077. doi: 10.3390/ma15093077 (PMC9102582; doi:10.3390/ma15093077)
Supplement: Supplementary file 1 [file materials-15-03077-s001.zip › materials-1675398-supplementary.pdf]

Supplementary Materials

Table S1. Experimental database of five input parameters (PET, SCMs, Flow, CS_{1d} and CS_{7d}) to evaluate the 28-d compression strength (CS_{28d}) of waste PET/SCM stabilized cementitious grout

	Input parameters					Output
S. No	PET (%)	SCMs (%)	Flow (Sec)	CS_{1d} (MPa)	CS_{7d} (MPa)	**28-d CS (MPa)**
1	10	5	26.5	9.34	27.13	39.60
2	0	5	14	26.51	48.28	65.77
3	7.5	0	17.4	9.26	27.86	38.26
4	10	5	24.8	11.61	29.92	42.64
5	5	5	16	18.10	32.94	54.31
6	5	5	16.3	17.86	37.46	54.26
7	0	0	12.7	32.16	37.54	57.91
8	2.5	5	14.92	20.84	42.07	63.17
9	10	10	28.1	8.39	27.43	39.37
10	5	0	14.6	12.80	25.31	39.64
11	5	5	15.6	20.12	36.47	58.30
12	5	10	21.5	19.83	37.38	60.60
13	2.5	0	13	13.90	28.14	41.35
14	0	10	17.2	33.32	50.97	69.41
15	5	10	22	21.82	42.57	62.38
16	5	10	20	17.87	41.29	57.32
17	2.5	5	15.8	21.58	37.68	61.75
18	2.5	0	12.5	12.00	31.82	42.50
19	10	5	24.3	13.34	26.46	39.14
20	5	0	15	10.38	30.82	41.35
21	0	0	12.4	28.22	40.40	54.18
22	10	0	21.6	7.56	25.60	39.40
23	0	5	13.04	25.34	51.02	63.86
24	7.5	5	20	15.14	29.25	53.45
25	0	10	17.8	30.51	55.05	71.12
26	7.5	5	19.4	13.51	28.79	49.47
27	7.5	0	18	8.44	26.04	40.60
28	0	5	13.45	27.70	44.98	66.58
29	0	0	12.6	30.12	42.25	58.41
30	7.5	0	18.2	10.86	22.96	37.91
31	0	10	16.9	30.74	49.64	66.83
32	10	10	26.26	14.01	29.82	43.37
33	2.5	5	15.2	24.65	38.65	59.16
34	7.5	5	19.6	17.36	33.36	51.98

35	5	0	15.4	13.88	27.91	43.87
36	10	0	22.5	6.35	19.79	33.64
37	10	0	23.4	5.64	19.19	36.02
38	10	10	28.6	9.49	32.29	43.79
39	2.5	0	14	16.53	30.80	45.89
40	10	5	26.5	17.65	28.43	42.60
41	0	5	14	26.51	48.28	65.77
42	7.5	0	16.9	11.07	30.36	44.05
43	10	5	25.55	17.56	30.74	45.36
44	5	5	15.8	24.79	42.88	60.14
45	5	5	16.3	19.84	40.45	63.05
46	0	0	12.7	32.16	37.54	57.91
47	2.5	5	14.9	26.32	48.10	65.41
48	10	10	28.1	14.63	31.87	46.78
49	5	0	14.3	16.42	32.03	42.55
50	5	5	15.3	22.41	46.41	59.97
51	5	10	20.8	22.97	42.74	62.17
52	2.5	0	13.3	16.30	34.91	48.65
53	0	10	17.2	33.32	50.97	69.41
54	5	10	22	21.57	45.61	63.47
55	5	10	19.6	25.87	44.06	66.91
56	2.5	5	15.05	23.74	44.74	60.84
57	2.5	0	12.5	18.71	39.77	45.90
58	10	5	24.6	20.14	32.14	46.50
59	5	0	14.85	13.07	33.88	44.42
60	0	0	12.4	28.22	40.40	54.18
61	10	0	21.6	8.04	25.41	39.87
62	0	5	13.04	25.34	51.02	63.86
63	7.5	5	20	18.04	34.14	59.14
64	0	10	17.8	30.51	55.05	71.12
65	7.5	5	19.9	19.48	38.09	56.08
66	7.5	0	18	10.57	31.48	40.88
67	0	5	13.45	27.70	44.98	66.58
68	0	0	12.6	30.12	42.25	58.41
69	7.5	0	17.45	13.81	29.41	40.55
70	0	10	16.9	30.74	49.64	66.83
71	10	10	25.85	18.42	34.62	51.25
72	2.5	5	15.2	22.31	43.25	62.98
73	7.5	5	19.8	21.36	34.68	60.01
74	5	0	15.4	14.93	36.60	46.92
75	10	0	21.8	6.89	27.16	41.09

76	10	0	21.7	9.37	22.74	37.26
77	10	10	26.975	15.63	30.61	47.98
78	2.5	0	14.1	20.24	37.55	50.71
79	5	10	13	17.13	39.36	59.08
80	0	5	11.375	29.08	48.24	65.42
81	7.5	0	17.4	9.26	27.86	38.26
82	5	10	12.725	18.26	41.71	60.09
83	10	5	18.4	6.91	26.29	46.15
84	10	5	18.2	9.61	28.05	44.69
85	0	0	12.7	32.16	37.54	57.91
86	5	5	13.8	18.03	33.84	51.11
87	10	10	16	10.77	30.87	51.18
88	5	0	14.6	12.80	25.31	39.64
89	10	5	18.6	9.06	30.87	43.07
90	7.5	10	15.7	12.29	36.34	52.63
91	2.5	0	13.9	13.90	28.14	41.35
92	0	10	9.3	28.32	58.81	77.35
93	7.5	10	15.8	12.90	32.95	53.21
94	7.5	10	15.6	15.70	31.74	57.20
95	5	5	13.5	15.76	39.30	57.44
96	2.5	0	13.8	12.00	31.82	42.50
97	5	10	12.45	20.18	37.49	55.74
98	5	0	14.4	10.38	30.82	41.35
99	0	0	12.4	28.22	40.40	54.18
100	10	0	19.42	7.56	25.60	39.40
101	0	5	11.5	24.48	50.00	63.13
102	2.5	10	13.7	20.69	40.88	66.10
103	0	10	9.2	32.05	57.46	82.54
104	2.5	10	13.4	18.20	40.17	62.91
105	7.5	0	18	8.44	26.04	40.60
106	0	5	11.25	26.14	47.33	67.69
107	0	0	12.6	30.12	42.25	58.41
108	7.5	0	17.7	10.86	22.96	37.91
109	0	10	9.1	30.98	61.81	81.45
110	10	10	15.8	8.93	28.13	49.61
111	5	5	13.2	15.30	38.59	54.51
112	2.5	10	13.1	22.74	43.83	64.54
113	5	0	14.2	13.88	27.91	43.87
114	10	0	19.52	6.35	19.79	33.64
115	10	0	19.47	5.64	19.19	36.02
116	10	10	15.9	9.89	31.13	52.66

117	2.5	0	14	16.53	30.80	45.89
118	5	10	12.5	25.61	45.45	66.82
119	0	5	11.525	29.08	48.24	65.42
120	7.5	0	17.4	11.07	30.36	44.05
121	5	10	12.475	22.65	49.96	62.25
122	10	5	18.46	10.04	34.14	50.04
123	10	5	18.32	14.31	29.64	48.68
124	0	0	12.7	32.16	37.54	57.91
125	5	5	13.8	22.08	43.98	63.47
126	10	10	15.6	14.44	31.82	54.63
127	5	0	14.6	16.42	32.03	42.55
128	10	5	18.6	12.37	30.47	52.55
129	7.5	10	15.5	17.96	36.93	58.95
130	2.5	0	14.05	16.30	34.91	48.65
131	0	10	9.4	28.32	58.81	77.35
132	7.5	10	15.4	18.14	35.22	59.63
133	7.5	10	15.6	17.73	38.41	56.43
134	5	5	13.95	22.65	48.98	60.47
135	2.5	0	14.1	18.71	39.77	45.90
136	5	10	12.45	22.36	46.83	67.16
137	5	0	14.45	13.07	33.88	44.42
138	0	0	12.4	28.22	40.40	54.18
139	10	0	19.1	8.04	25.41	39.87
140	0	5	11.8	24.48	50.00	63.13
141	2.5	10	13.6	27.32	54.62	68.73
142	0	10	9.25	32.05	57.46	82.54
143	2.5	10	13.35	28.22	49.24	65.27
144	7.5	0	16.9	10.57	31.48	40.88
145	0	5	11.25	26.14	47.33	67.69
146	0	0	12.6	30.12	42.25	58.41
147	7.5	0	17.15	13.81	29.41	40.55
148	0	10	9.1	30.98	61.81	81.45
149	10	10	15.8	14.40	35.12	58.34
150	5	5	14.1	19.98	46.92	60.62
151	2.5	10	13.1	29.21	53.41	67.36
152	5	0	14.3	14.93	36.60	46.92
153	10	0	19.52	6.89	27.16	41.09
154	10	0	19.31	9.37	22.74	37.26
155	10	10	15.7	14.42	32.48	53.89
156	2.5	0	14	20.24	37.55	50.71
